# Bibliometric analysis of recent research on the association between TRPV1 and inflammation

**DOI:** 10.1080/19336950.2023.2189038

**Published:** 2023-03-15

**Authors:** Pan Xu, Ru-Ru Shao, Yuan He

**Affiliations:** Department of Oral Medicine, Stomatological Hospital and Dental School of Tongji University, Shanghai Engineering Research Center of Tooth Restoration and Regeneration, Shanghai, China

**Keywords:** TRPV1 channel, inflammation, bibliometrics, CiteSpace, Vosviewer

## Abstract

TRPV1 channel is a sensitive ion channel activated by some noxious stimuli and has been reported to change many physiological functions after its activation. In this paper, we present a scientometric approach to explore the trends of the association between TRPV1 channel and inflammation and our goal is to provide creative directions for future research. The related literature was retrieved from Web of Science Core Collection and then analyzed by CiteSpace and VOSviewer. A total of 1533 documents were screened. The most productive country, institution, journal, author, cited journal, cited author, and references were the United States, University of California, San Francisco, Pain, Lu-yuan Lee, Nature, Michael J. Caterina, and Caterina MJ (Science, 2000), respectively. The most influential country and institution were Switzerland and University of California, San Francisco, respectively. The cooperation among countries or institutions was extensive. Amounts of documents were distributed in molecular, biology, genetics. TRPV1-associated neurons, neuropeptides, neuropathic pain, neuroinflammation, and neurogenic inflammation were mainly hotspots in this field. The research has presented valuable data about previous studies in the link of TRPV1 channel and inflammation.

Transient Receptor Potential (TRP) channels belong to nonselective cation channels and were divided into six subfamilies including TRPC (canonical), TRPV (vanilloid), TRPM (melastatin), TRPML (mucolipin), TRPP (polycystin), and TRPA1 (ankyrin) channels in human [1]. Among these channels, TRPV1 channel has been widely studied. TRPV1 also called as capsaicin receptor. As the name suggests, TRPV1 is very sensitive to capsaicin and the latter activates TRPV1 channel by acting on binding sites [2,3]. A well-known researcher in TRPV1 field is David Julius, one of 2021 Nobel Prize in Physiology or Medicine winners. His pioneer work has contributed to our present understanding of this channel. Most strikingly, his lab isolated a functional cDNA encoding a capsaicin receptor from sensory neurons named VR1 (that is TRPV1) using an expression cloning strategy based on calcium influx [2], which threw light on the nature of TRPV1 channel. Based on hydrophilicity analysis, TRPV1 channel was firstly reported to be a polytopic protein containing six transmembrane domains with an additional short hydrophobic stretch between transmembrane regions 5 and 6 [2]. Afterward, he participated in unraveling the structure of TRPV1 channel by single particle electron cryo-microscopy, which is very important for the study of its function [4].

Many studies have shown that TRPV1 channel was activated by several exogenous (noxious heat (>~43°C) [2], acidic pH [5], protons [6], toxin [7]) and endogenous agonists (bradykinin [8], lipid [9], diacylglycerol [10]). For this, TRPV1 activation in neuronal and non-neuronal cells could exert various physiological functions and has been extensively studied by researchers from different scientific fields. TRPV1 has been widely expressed in neurons, especially neurons of small to medium size in the dorsal root and trigeminal ganglia [2], and it was responsible for nociception transduction and inflammatory thermal hyperalgesia as a thermosensor [11]. Also, TRPV1 channel was functionally expressed in non-neuronal cells. For instance, TRPV1 in vascular smooth muscle cells could mediate vasoconstriction of the resistance arteries [12]. Activated TRPV1 signaling increased cell proliferation of gingival epithelial cells [13]. *Prevotella melaninogenica*, a pathogenic bacteria that causes a variety of anaerobic infections, was found to be able to activate the TRPV1 channel in our study. Additionally, the results showed that TRPV1 activation promoted inflammation, which raised the possibility of a link between the production of inflammation and TRPV1 channel activation [14].

Inflammatory response is a complicated physiological and pathological processes from trigger, physiological purpose to pathological consequences [15]. More and more researchers have focused on the association between TRPV1 channel and inflammation. Many reports have revealed that TRPV1 could trigger inflammatory response by releasing inflammatory neuropeptides substance P (SP) and calcitonin gene-related peptide (CGRP) in neuronal cells [16]. SP and CGRP exerted their pro-inflammatory role by interacting their receptors in many types of cells including T lymphocytes, macrophages, mast cells [17–19], thus arousing immune responses. During inflammation, TRPV1 channel participated in the process of heat, mechanical, and cold hyperalgesia [20]. Aberrant expression and activation of TRPV1 have been reported to be involved in inflammatory diseases such as oral lichen planus (OLP) [21], psoriasis [22], atopic dermatitis [23], airway inflammation [24], inflammatory bowel diseases (IBD) [25], and pancreatitis [26]. In non-neuronal cells, TRPV1 activation by capsaicin in keratinocytes promoted the release of interleukin-8 and prostaglandin E2, resulting in inflammation [27]. TRPV1/CGRP pathway induced inflammatory response in Hacat and THP-1 cells challenged with *Propionibacterium acnes* [28]. Considering its important role both in neurons and non-neuronal cells, some scholars proposed that TRPV1 may be a keystone for neurogenic inflammation [29–31].

Bibliometric analysis was an effective method to review prominent publications, to analyze current research and predict research trends in the future [32]. It can be useful for researchers to have access to data on the frequency and distribution of countries/regions, institutions, authors, journals, co-cited authors, and journals in a certain field. Bibliometric analysis has become popular in recent years across a number of medical specialties, including rehabilitation medicine [33], neurology [34], anesthesiology [35], respiratory system [36], and oral medicine [37]. A free computer tool called VOSviewer makes it simple to create and analyze bibliometric maps [38]. Another bibliometric tool for evaluating and visualizing trends and hotspots in a knowledge domain is CiteSpace [39], which is widely used in bibliometric analysis. Although the TRPV1 channel and inflammation are popular topics in several research areas, no bibliometric studies have yet established a connection between the two. Here, we explored the trends and potential association between TRPV1 channel and inflammation by a bibliometric approach, aiming to provide a scientific collaboration network and new directions for future research.

## Data collection

A search for related articles up to 2021 was conducted in the Web of Science Core Collection (WOSCC). The data were downloaded directly from the database as secondary data without further animal experiments, and thus, no ethical approval was required. The data search was conducted on 15 September 2022 and collected in 1 day to avoid any potential bias due to the daily updating of the database. The search formula was TS = [TS = (“Inflammation” OR “Inflammations” OR “Innate Inflammatory Response” OR “Inflammatory Response, Innate” OR “Innate Inflammatory Responses”)] AND [TS = (“TRPV1” OR “TRPV-1” OR “vanilloid receptor 1” OR “transient receptor potential cation channel subfamily V member 1 protein” OR “transient receptor potential vanilloid 1” OR “transient receptor potential V1” OR “capsaicin receptor”)]. The document language was set to English. In total, 1533 publications were analyzed. The retrieving process was shown in **Figure S1**.

## Data analysis

CiteSpace software (6.1 R3) and VOSviewer were used to perform a bibliometric analysis including authors, keywords, countries/regions, references, co-cited authors, and journals. Visualization maps consist of nodes and links. Different nodes represent elements such as an author, keyword, country/region, reference, journal, and links between nodes represent collaboration or co-occurrence or co-citations among them. The color of nodes and lines represents different years. The parameters of CiteSpace were set as follows: time slicing (1993–2021), years per slice (1), term source (all selection), node type (choose 1 at a time), pruning (pathfinder) and visualization (cluster view-static, show merged network). For country/region, institution, keyword, cited author, and journal, the TOP N was set 50. For author, the TOP N was set 10. The purple circle represented centrality, nodes with high centrality were usually considered as pivotal points or key points in a specific field [40,41]. The distribution of countries and institutions was drawn using Microsoft Excel 2019 (Microsoft Office for Windows, USA). See **Supplementary material Table S1** for details.

## General information and annual publication output

**Figure 1.**
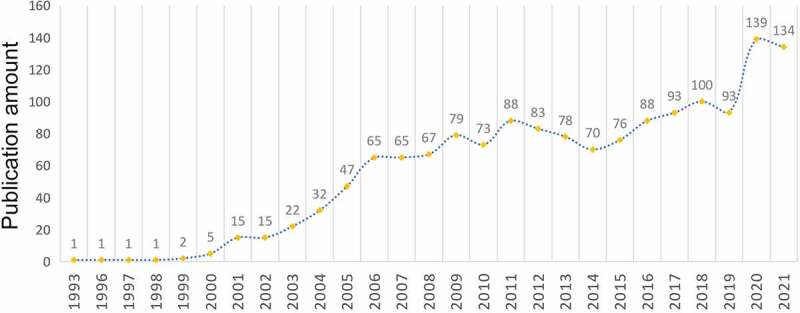
Analysis of annual publications. The numbers of annual publications on TRPV1 channel and inflammation.

We searched a total of 1925 records based on the search terms, of which 1533 met the requirements including 1283 articles and 250 reviews. On the whole, the number of published articles presented a steady upward trend from 1993 to 2021 and the published documents in 2021 increased by 134 times compared to that in 1993 with an average annual publication of 52.9 documents and a compound annual growth rate of 18.4% (Figure 1). The increase in the number also shows that there have been more and more researches in this field and scholars paid more attention to it.

## Active countries/regions and institutions

**Figure 2.**
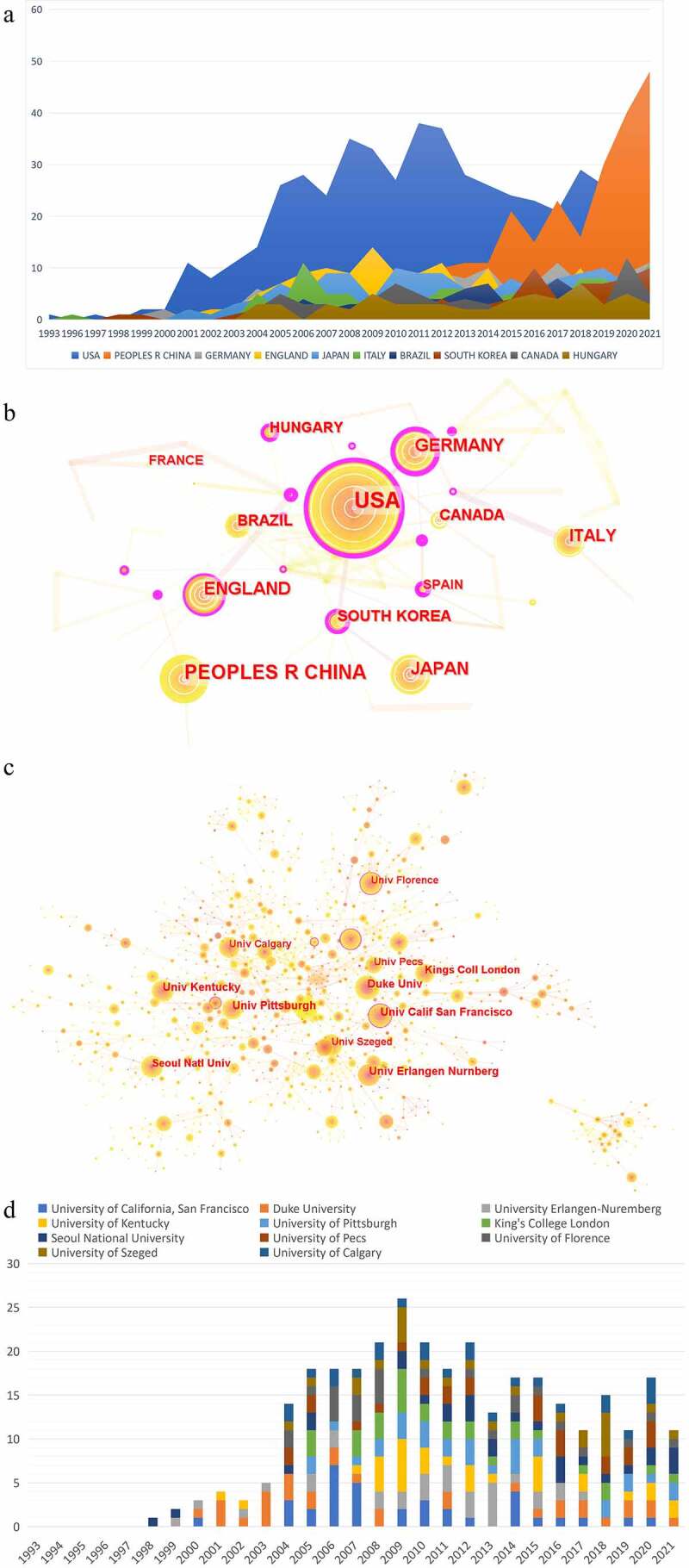
Analysis of country and institution. (a) The publication distribution related to TRPV1 channel and inflammation of top 10 countries; (b) Network map of the main countries of publications by CiteSpace; (c) Network map of institutions in the research about TRPV1 channel and inflammation; (d) The publication distribution related to TRPV1 channel and inflammation of top 11 institutions. The purple round represents centrality. Nodes with high centrality are usually regarded as pivotal points in the field.

Many countries and institutions contributed to this research. We analyzed the countries and institutions of publications over the past 29 years (1993–2021) including a total of 62 countries/regions, and the cooperation between them constituted 272 links. As for countries/regions, the United States (USA) (*n* = 548) dominated this field, followed by China (*n* = 257), Germany (*n* = 142), England (*n* = 139), and Japan (*n* = 138) in publication amount. From a centrality perspective, Switzerland (centrality = 0.69) topped the list, followed by Romania (centrality = 0.63), Finland (centrality = 0.55), Germany (centrality = 0.48), and the United States (centrality = 0.44) (Table 1). Among these, there were close cooperation among the United States, Japan, South Korea, and Germany (Figure 2a and b). In terms of institutions, there were 1509 nodes and 3753 links. Among these, University of California, San Francisco (*n* = 35) ranked first, followed by Duke University (*n* = 33), University Erlangen-Nuremberg (*n* = 32), University of Kentucky (*n* = 32), and University of Pittsburgh (*n* = 31) (Table 2). Among these institutions, there was close collaboration (Figure 2c and d). As for influence, University of California, San Francisco (centrality = 0.18) ranked first, followed by University of Florence (centrality = 0.12), National Cancer Institute (centrality = 0.12), New York University (centrality = 0.11), and Consiglio Nazionale delle Ricerche (centrality = 0.10) (Table 2). Of particular note is that the communication and cooperation among countries or institutions were very necessary.

**Table 1.** Top 10 countries/regions of publications and centrality in TRPV1 channel and inflammation field.

	Publications				Centrality		
Rank	Country/region	Frequency (*N* = 2065)	Percentage		Rank	Country/region	counts
1	USA	548	26.54%		1	Switzerland	0.69
2	Peoples R China	257	12.45%		2	Romania	0.63
3	Germany	142	6.88%		3	Finland	0.55
4	England	139	6.73%		4	Germany	0.48
5	Japan	138	6.68%		5	USA	0.44
6	Italy	101	4.89%		6	South Korea	0.36
7	Brazil	74	3.58%		7	Portugal	0.34
8	South Korea	73	3.54%		8	Austria	0.25
9	Canada	68	3.29%		9	Russia	0.24
10	Hungary	60	2.91%		10	Sweden	0.22

**Table 2.** Top 10 institutions of publications and centrality in TRPV1 channel and inflammation field.

	Rank	Institution	Frequency (*N* = 3395)	Percentage
Amounts	1	University of California, San Francisco	35	1.03%
	2	Duke University	33	0.97%
	3	University Erlangen-Nuremberg University of Kentucky	32	0.94%
	5	University of Pittsburgh	31	0.91%
	6	King’s College London	29	0.85%
	7	Seoul National University	28	0.82%
	8	University of Pecs	26	0.77%
	9	University of Florence	25	0.74%
	10	University of Szeged University of Calgary	24	0.71%
Centrality	1	University of California, San Francisco	0.18	
	2	University of Florence National Cancer Institute	0.12	
	4	New York University	0.11	
	5	Consiglio Nazionale delle Ricerche	0.10	
	6	University of California,Davis Indiana University	0.09	
	8	Charite University Hospital Kaohsiung Medical University	0.08	

## Active journals and co-cited journal

**Figure 3.**
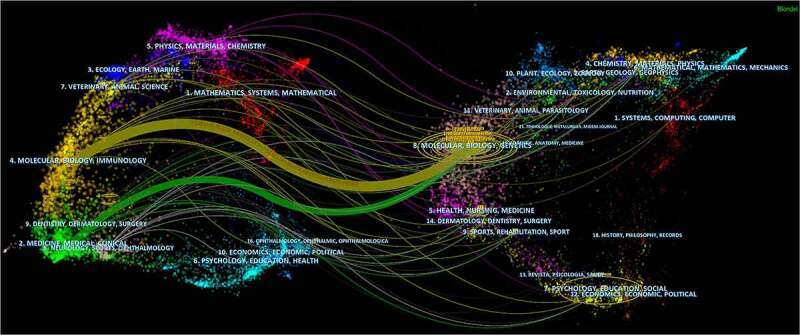
The dual-map overlay of journals related to TRPV1 channel and inflammation research. Colored curves symbolized paths of references, where each curve originated from a citing journal on the left side of the dual-map and pointed at a cited journal on the right side.

Dual-map overlay map of journals showed that a large number of the papers were distributed in molecular, biology, genetics, and some were health, nursing, medicine, and dermatology, dentistry and surgery (Figure 3).

**Figure 4.**
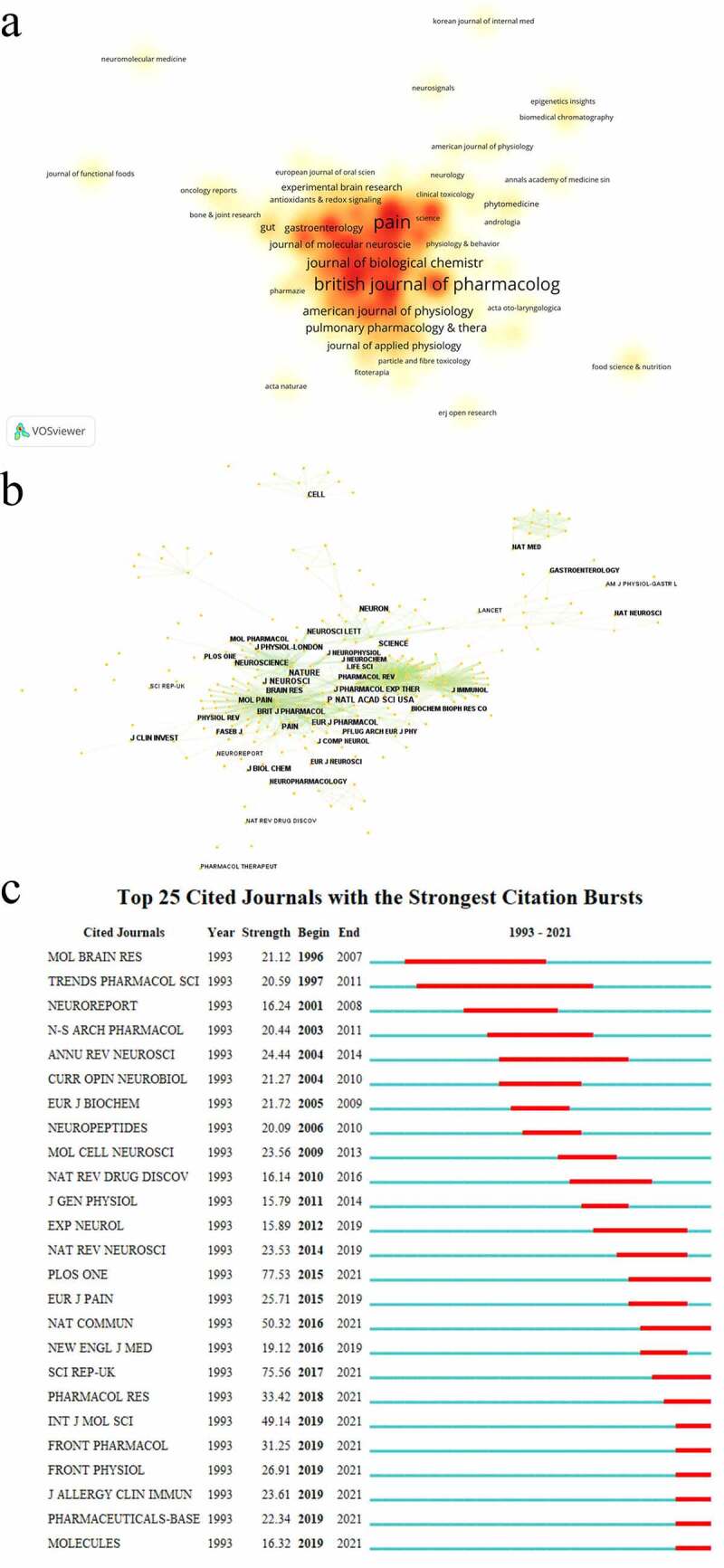
Analysis of journals and co-cited journals in TRPV1 channel and inflammation. (a) the distribution density of journals in the research by VOSviewer; (b) Network map of co-cited journals in the research by CiteSpace; (c) Top 25 cited journals with the strongest citation bursts in the research by CiteSpace.

Totally, 499 journals published documents about TRPV1 and inflammation and journals were shown in Figure 4a. Among these, *Pain* (*n* = 50) was the most frequent journal in this field, followed by *Molecular Pain* (*n* = 45), *Journal of Neuroscience* (*n* = 42), *British Journal of Pharmacology* (*n* = 40) and *Neuroscience* (*n* = 32). The most citation was from *Journal of Neuroscience* (*n* = 4689) with the citations per paper (111.64). The impact factor of top 10 journals was from 3.37 to 9.473 and JCI quartile included 40% Q1, 40% Q2 and 20% Q3 (Table 3). Some documents were published in well-known journals, such as *Nature* (*n* = 3), *Cell* (*n* = 3) and *Science* (*n* = 1).

**Table 3.** Top 10 productive journals in TRPV1 channel and inflammation field.

Rank	Journal	Counts	Citations	Citations per paper	Impact factors (2021)	JCI quartile (2021)
1	Pain	50	2644	52.88	7.926	Q1
2	Molecular Pain	45	1996	44.36	3.370	Q3
3	Journal of Neuroscience	42	4689	111.64	6.709	Q1
4	British Journal of Pharmacology	40	2329	58.23	9.473	Q1
5	Neuroscience	32	1408	44	3.708	Q3
6	European Journal of Pharmacology	28	1250	44.64	5.195	Q2
6	Scientific Reports	28	618	22.07	4.996	Q2
7	Journal of Pharmacology and Experimental Therapeutics	25	1871	74.84	4.441	Q2
7	Plos One	25	976	39.04	3.752	Q2
7	International Journal of Molecular Sciences	25	194	7.76	6.208	Q1

The network of co-cited journals included 340 nodes and 1701 links (Figure 4b) and the top 10 was shown in Table 4. The most co-cited journal was Nature with 1123 citations, followed by Journal of *Neuroscience* (*n* = 1047), *PNAS* (*n* = 992), *Pain* (*n* = 921) and *British Journal of Pharmacology* (*n* = 863). The articles published in those top journals reflect the basis of the research field. As for the burst detection, *Annual Review of Neuroscience* showed the strongest burst (strength = 24.44) from 2004–2014, *Plos one* showed the strongest burst (strength = 77.53) from 2015–2021 (Figure 4c).

**Table 4.** Top 10 productive cited journals in TRPV1 channel and inflammation field.

Rank	Journal	Citation counts	Impact factors (2021)	JCI quartile (2021)
1	Nature	1123	69.504	Q1
2	Journal of Neuroscience	1047	6.709	Q1
3	Proceedings of the National Academy of Sciences of the United States of America (PNAS)	992	12.779	Q1
4	Pain	921	7.926	Q1
5	British Journal of Pharmacology	863	9.473	Q1
6	Journal of Biological Chemistry	822	5.486	Q2
7	Neuroscience	788	3.708	Q3
8	Neuron	775	18.688	Q1
9	Science	730	63.798	Q1
10	European Journal of Pharmacology	692	5.195	Q2

## Active authors and cited authors

**Figure 5.**
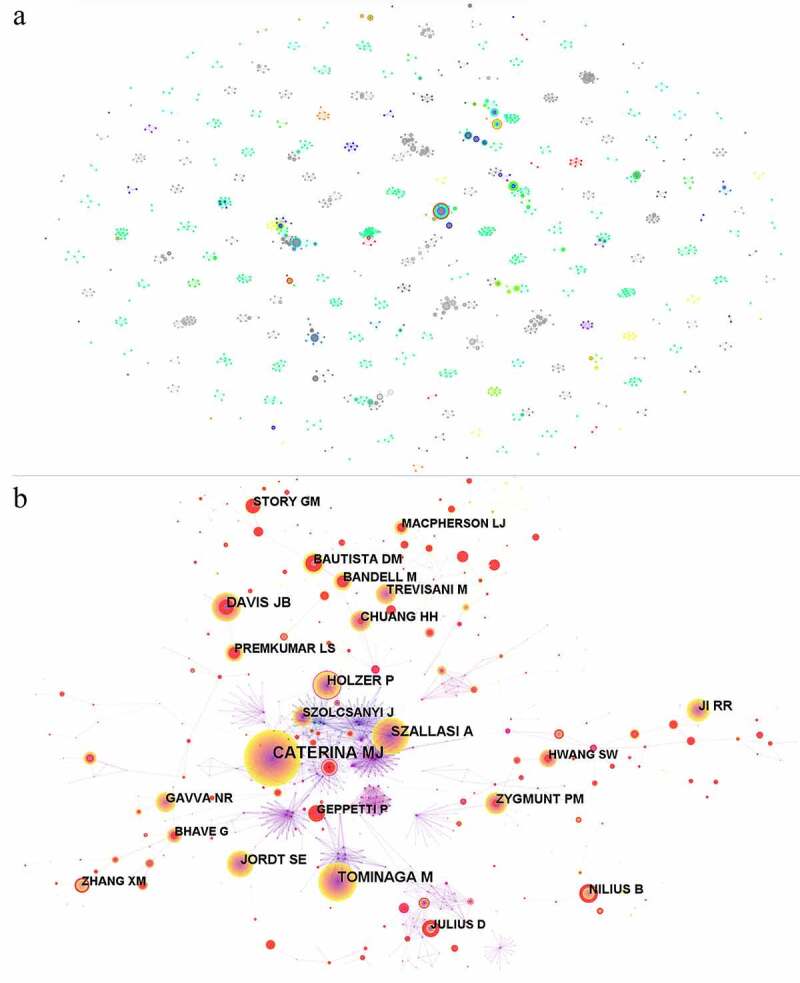
Analysis of authors and co-cited authors in TRPV1 channel and inflammation. Network map of authors (a) and cited authors (b) by CiteSpace.

Total 7678 authors contributed to the related documents in TRPV1 and inflammation, 1237 of which were shown in the author-network map (Figure 5a). The map showed that the most prolific authors. As shown in Table 5, Lu-yuan Lee, a well-known professor from Department of Physiology, University of Kentucky Medical Centre in USA, participated in the publication of 18 articles, ranking first, as the most productive author, has made remarkable achievements on the research about TRPV1 channel and sensory neurons. In his highest cited related articles (*n* = 88), he illustrated that allergen-induced chronic inflammation in airway could promote the sensitivity to capsaicin of vagal bronchopulmonary myelinated afferents, which probably resulted from an increased expression of TRPV1 in these sensory nerves [42], which emphasized the promotion of inflammation for TRPV1 activation. From the Figure 5a, it was seen that the collaborations among authors was little.

**Table 5.** Top 10 authors of publications in TRPV1 channel and inflammation field.

Authors	Document accounts	Article with the highest citation	Citations
Lu-yuan Lee	18	Altered expression of TRPV1 and sensitivity to capsaicin in pulmonary myelinated afferents following chronic airway inflammation in the rat	88
Zsuzsanna Helyes	12	Evidence for the role of lipid rafts and sphingomyelin in Ca2±gating of Transient Receptor Potential channels in trigeminal sensory neurons and peripheral nerve terminals	58
Pierangelo Geppetti	12	4-Hydroxynonenal, an endogenous aldehyde, causes pain and neurogenic inflammation through activation of the irritant receptor TRPA1	554
Peter W Reeh	12	The Molecular Basis for Species-specific Activation of Human TRPA1 Protein by Protons Involves Poorly Conserved Residues within Transmembrane Domains 5 and 6	73
Brian M Davis	9	TRPV1 unlike TRPV2 is restricted to a subset of mechanically insensitive cutaneous Nociceptors responding to heat	118
Janos Szolcsanyi	8	Evidence for the role of lipid rafts and sphingomyelin in Ca2±gating of Transient Receptor Potential channels in trigeminal sensory neurons and peripheral nerve terminals	58
Juliano Ferreira	7	Transient Receptor Potential Ankyrin 1 Receptor Stimulation by Hydrogen Peroxide Is Critical to Trigger Pain During Monosodium Urate-Induced Inflammation in Rodents	58
Narender R Gavva	7	Pharmacological blockade of the vanilloid receptor TRPV1 elicits marked hyperthermia in humans	350
Donna H Wang	7	Transient Receptor Potential Vanilloid Gene Deletion Exacerbates Inflammation and Atypical Cardiac Remodeling After Myocardial Infarction	60
Armen N Akopian	7	Activation of TRPV1 in the spinal cord by oxidized linoleic acid metabolites contributes to inflammatory hyperalgesia	164

For cited authors in these articles, a network was shown in Figure 5b including 691 nodes and 2664 links and the collaborations among them was visible. The most frequently cited author is Caterina MJ with a total of 880 citations followed by Tominaga M (*n* = 404), Szallasi A (*n* = 359), Davis JB (*n* = 299), and Jordt SE (*n* = 231) (Table 6). According to their articles, many great contributions has been made to the research on TRPV1 channel and inflammation. For example, the essential role of TRPV1 channel in pain sensation was proved [43]. It was also reported that the p38 MAPK activation in the DRG increased translation and transport of TRPV1 to the peripheral nociceptor terminal, leading to the maintenance of inflammatory hypersensitivity [44], which closely linked TRPV1 and inflammatory hypersensitivity, providing strong support and guidance for the subsequent exploration. Moreover, it was also identified that TRPV1 in concert with TRPA1 were required for bradykinin-evoked thermal hyperalgesia in inflammatory pain [45].

**Table 6.** Top 10 cited authors of publications in TRPV1 channel and inflammation field.

Rank	Cited authors	Citations
1	Michael J. Caterina	880
2	Makoto Tominaga	404
3	Arpad Szallasi	359
4	John B. Davis	299
5	Sven-Eric Jordt	231
6	Peter Holzer	207
7	Ru-Rong Ji	203
8	Diana M Bautista	197
9	Huai-Hu Chuang	175
10	Narender R Gavva	170

## Co-citation analysis

**Figure 6.**
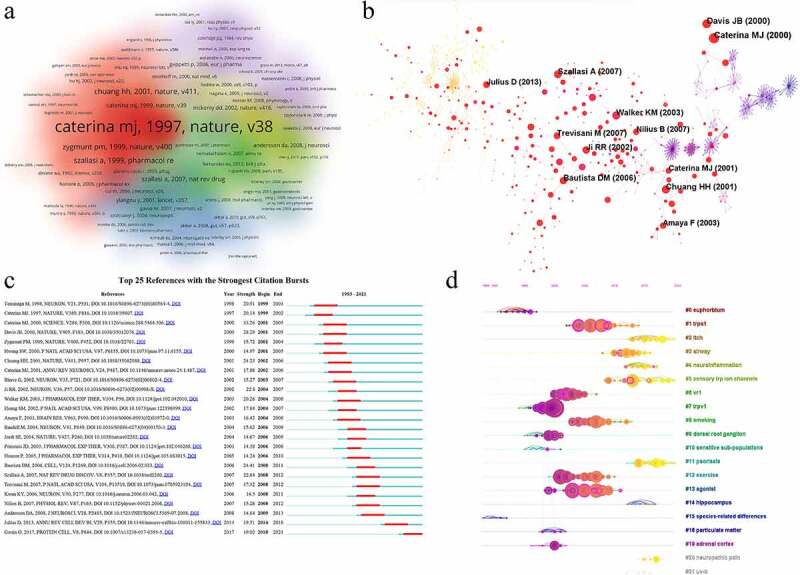
Analysis of co-cited references. (a) the density of co-cited references in TRPV1 channel and inflammation by VOSviewer; (b) the network map of co-cited references in TRPV1 channel and inflammation by CiteSpace; (c) Top 25 references with the strongest citation bursts in this research; (d) the timeline view of the 19 clusters.

We performed an analysis of references using CiteSpace and VOSviewer. A total of 47,472 documents were included. The top three cited references including Caterina MJ (Science, 2000), Davis JB (Nature, 2000) and Bautista DM (Cell, 2006) and has more than 60 citations (Table 7). The co-citation density and network were shown in Figure 6a and b. For burst detection, strong burst was shown in Caterina MJ (Science, 2000) and Davis JB (Nature, 2000) in the early phase (2001–2005), Bautista DM (Cell, 2006) in the middle phase (2008–2011) and Gouin O (Protein Cell, 2017) in the late phase (2018–2021) (Figure 6c). Timeline of cited references were generated by CiteSpace and 19 clusters in regions with different colors comprised of #0 euphorblum, #1 TRPA1, #2 itch, #3 airway, #4 neuroinflammation, #5 sensory TRP ion channels, #6 vr1, #7 TRPV1, #8 smoking, #9 dorsal root ganglion, #10 sensitive sub-populations, #11 psoriasis, #12 exercise, #13 agonist, #14 hippocampus, #15 species-related differences, #16 particulate matter, #19 adrenal cortex, #20 neuropathic pain, and #21 UV-b. It was found that documents related to TRPA1, smoking, exercise, and agonists were gathered in 2000–2010, documents related to itch, airway, neuroinflammation, psoriasis, and neuropathic pain were concentrated on 2010–2020 (Figure 6d). Combined analysis of burst detection and timeline revealed that different topics of references were in different phases. In the early exploration, some fundamental findings about TRPV1 channel in inflammation have been published. TRPV1 was increased after inflammation and it was required for inflammatory thermal hyperalgesia [11,43,44,46,47], which revealed the importance of TRPV1 in regulation of inflammatory pain. The synergistic role of TRP channels (such as TRPV1 and TRPA1) in inflammation and pain was reported [48–50]. In recent years, TRPV1 was reported to mediates neuroinflammation in microglia [51] and regarded as a potential treatment target for neuropathic pain [52].

**Table 7.** Top 10 references with citations in TRPV1 channel and inflammation field.

Rank	Title	First author	Journal	Publication year	Citations	DOI
1	Impaired Nociception and Pain Sensation in Mice Lacking the Capsaicin Receptor	Caterina MJ	Science	2000	71	10.1126/science.288.5464.306
2	Vanilloid receptor-1 is essential for inflammatory thermal hyperalgesia	Davis JB	Nature	2000	61	10.1038/35012076
3	TRPA1 Mediates the Inflammatory Actions of Environmental Irritants and Proalgesic Agents	Bautista DM	Cell	2006	60	10.1016/j.cell.2006.02.023
4	The vanilloid receptor TRPV1: 10 years from channel cloning to antagonist proof-of-concept	Szallasi A	Nat Rev Drug Discov	2007	56	10.1038/nrd2280
5	Bradykinin and nerve growth factor release the capsaicin receptor from PtdIns(4,5)P_2_-mediated inhibition	Chuang HH	Nature	2001	54	10.1038/35082088
6	p38 MAPK Activation by NGF in Primary Sensory Neurons after Inflammation Increases TRPV1 Levels and Maintains Heat Hyperalgesia	Ji RR	Neuron	2002	50	10.1016/S0896–6273(02)00908-X
7	The VR1 antagonist capsazepine reverses mechanical hyperalgesia in models of inflammatory and neuropathic pain	Walker KM	J Pharmacol Exp Ther	2003	48	10.1124/jpet.102.042010
8	4-Hydroxynonenal, an endogenous aldehyde, causes pain and neurogenic inflammation through activation of the irritant receptor TRPA1	Trevisani M	PNAS	2007	43	10.1073/pnas.0705923104
9	TRPA1 Contributes to Cold, Mechanical, and Chemical Nociception but Is Not Essential for Hair-Cell Transduction	Kwan KY	Neuron	2006	40	10.1016/j.neuron.2006.03.042
9	The Vanilloid Receptor: A Molecular Gateway to the Pain Pathway	Caterina MJ	ANNU REV NEUROSCI	2001	40	10.1146/annurev.neuro.24.1.487

## Keyword co-occurrence and burst

**Figure 7.**
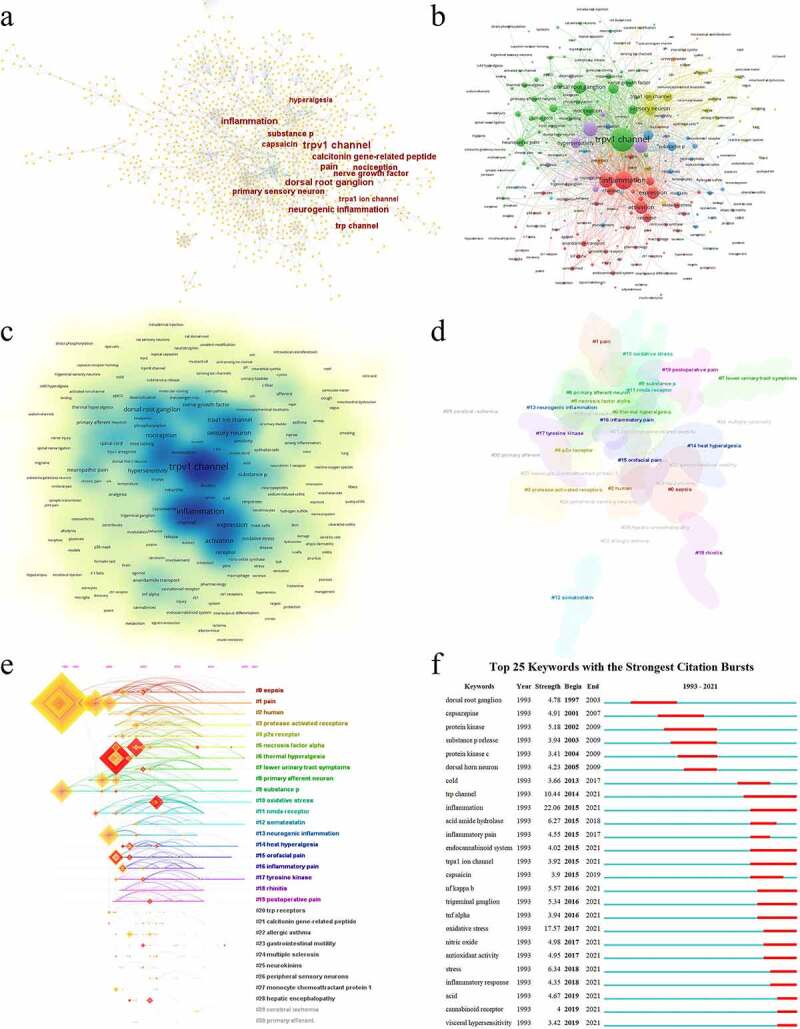
Analysis of keywords and burst detection. The network map of keywords in TRPV1 channel and inflammation by Citespace (a) and VOSviewer (b); (c) the density of keywords in TRPV1 channel and inflammation by VOSviewer; (d) Cluster visualization of the keyword map; (e) the timeline view of the clusters; (f) Top 25 keywords with the strongest citation bursts.

To some extent, the analysis of keywords could reflect the direction and interest of research in this field. Keyword network and density were generated by CiteSpace and VOSviewer (Figure 7a-c). The frequency of keywords can represent the trends of research. It was found that the most frequent keywords in TRPV1 and inflammation were TRPV1 channel (*n* = 858), inflammation (*n* = 351), dorsal root ganglion (*n* = 279), pain (*n* = 238), calcitonin gene-related peptide (*n* = 178), neurogenic inflammation (*n* = 173), substance p (*n* = 163), TRP channel (*n* = 156), capsaicin (*n* = 125), and nerve growth factor (*n* = 125) (Table 8). In the overlay network, 31 clusters were shown including #0 sepsis, #1 pain, #2 human, #3 protease-activated receptors, #4 p2× receptor, #5 necrosis factor, #6 thermal hyperalgesia, #7 lower urinary tract symptoms, #8 primary afferent neuron, #9 substance p, #10 oxidative stress, #11 nmda receptor, #12 somatostatin, #13 neurogenic inflammation, #14 heat hyperalgesia, #15 orofacial pain, #16 inflammatory pain, #17 tyrosine kinase, #18 rhinitis, #19 postoperative pain, #20 TRP receptors, #21 calcitonin gene-related peptide, #22 allergic asthma, #23 gastrointestinal mobility, #24 multiple sclerosis, #25 neurokinins, #26 peripheral sensory neurons, #27 monocyte chemoattractant protein 1, #28 hepatic encephalopathy, #29 cerebral ischemia, and #30 primary afferent (Figure 7d). As well, the timeline view presented these clusters over time and it was shown that many influential research subjects occurred in the early phase such as pain, thermal hyperalgesia, and substance p (Figure 7e). The strongest keyword citation burst analysis helped to illustrate the research’s frontiers. For burst detection, inflammation (strength = 22.06, 2015–2021), oxidative stress (strength = 17.57, 2017–2021), and TRP channel (strength = 10.44, 2014–2021) showed strong burst (Figure 7f), which reflected the recent research trends in this field.

**Table 8.** Top 20 keywords in terms of frequency in TRPV1 channel and inflammation field.

Rank	Keywords	Frequency	Rank	Keywords	Frequency
1	TRPV1 channel	858	11	nociception	122
2	inflammation	351	12	primary sensory neuron	117
3	dorsal root ganglion	279	13	TRPA1 ion channel	113
4	pain	238	14	hyperalgesia	111
5	calcitonin gene-related peptide	178	15	spinal cord	78
6	neurogenic inflammation	173	16	oxidative stress	74
7	substance p	163	17	protein kinase c	69
8	TRP channel	156	18	primary afferent neuron	68
9	capsaicin	125	19	thermal hyperalgesia	61
9	nerve growth factor	125	20	inflammatory pain	51

On the whole, the researchers mainly focused on TRPV1-associated exploration, such as TRPV1 agonist capsaicin, neuropeptides (substance P, CGRP), some other ion channels and neurogenic inflammation and neuropathic pain. From a perspective of burst detection and overlay network, we predicted the following five important hotspots in this field:

1. TRPV1 expression: As a sensitive ion channel, many studies showed that TRPV1 was expressed in dorsal root ganglion (DRG). Blockade of TRPV1 in DRG was considered as a potential method in cardioprotection [53], irritable bowel syndrome [54], psoriasis [22], prostatitis [55], neuropathic pain [56], cystitis [57].
2. Transient Receptor Potential Ankyrin 1 (TRPA1) ion channel: The structure of TRPA1 is similar with TRPV1 [58], and they shared many similarities in the mechanism of oxidative stress [59], inflammation [60], and pain [61]. However, they were different. It was reported that 97% (100/103) of TRPA1-positive neurons also express TRPV1, while 30% (100/336) of TRPV1-positive neurons express TRPA1 [62]. Although they were both temperature-sensitive channels, TRPA1 was sensitive to cold temperature instead of heat like TRPV1 [63].
3. Neuropathic pain: TRPV1 could induce neuropathic pain in many situations. For example, as a target of miR-338-3p, TRPV1 induced neuropathic pain via interacting with N-terminal EF-hand Ca^2+^-binding proteins 2 [64]. Also, Pirt together with TRPV1 is involved in the regulation of neuropathic pain [65]. TRPV1 could affect their intrinsic electrical properties and synaptic strength in cortex of mice suffering from neuropathic pain [66]. Moreover, blocking TRPV1 channel could be considered as a way in clinical treatment. Zinc suppressed both acute nociception and chemotherapy-induced chronic neuropathic pain through inhibiting TRPV1 [67]. As well, noopept, a nootropic dipeptide, attenuated diabetes-mediated neuropathic pain through inhibition of TRPV1 channel [68].
4. Neurogenic inflammation: Neurogenic inflammation is a complex process through the release of the neuropeptides CGRP and SP and subsequent vascular and non-vascular inflammatory responses including vasodilation, plasma extravasation, edema, and mast cell degranulation [69]. TRPV1 could induce neurogenic inflammation by promoting the release of neuropeptides SP and CGRP upon activation [30]. In airways, ethanol could stimulate airway sensory nerves through TRPV1 channels, resulting in the release of sensory neuropeptides and the consequent inflammatory responses [70]. In trigeminal sensory neurons, the activation of TRPV1 channels lead to CGRP release and activate NF-κB nuclear translocation, contributing to the inflammation [16], and inflammatory mediators could activate TRPV1 ion channels, leading to the release of neuropeptides, thereby inducing or enhancing neurogenic inflammatory processes [71].
5. Substance P (SP): As mentioned above, TRPV1 activation could result in the release of neuropeptide substance P [72]. SP was expressed in many cell types such as neurons [73], immune cells (T cells, macrophages) [74,75] and exerted its pro-inflammatory role through interacting with its receptors [76]. SP could be regarded as a very important bridge for researchers to study the crosstalk between TRPV1 and inflammation.

## Recent research

With the deepening of research, more and more researchers have focused on the role of TRPV1 in treatment. Since 2022, nearly half of the literature has presented TRPV1 as a potential target or TRPV1 antagonists as potential therapeutics. A clinical trial showed that topical application of Asivatrep cream (TRPV1 antagonist) improved clinical signs and symptoms of atopic dermatitis [77]. Another TRPV1 antagonist, *cis-Jasmone* was reported to have a topical anti-inflammatory effect in skin inflammation [78]. On the other hand, as a potential target, TRPV1 could be regulated by some drug agents (Berberine [79], Shixiao San [80], plant extracts (*Lycium barbarum* Polysaccharides [81], Paeoniflorin [82], and electroacupuncture [83,84] for relieving pain and/or anti-inflammatory effect. In cancer therapy, TRPV1 hyperactivation promoted tumor initiation and progression and lead to alterations in tumor microenvironment during colorectal tumorigenesis. Due to this, TRPV1 was suspected to be a potential target for the immunotherapy of colorectal cancer [85]. The researchers also discovered a novel capsaicin-loaded CaCO3 nanoparticle for potential cancer treatment [86]. Additionally, the study about TRPV1 activation and pain still goes on. For example, the synthetic chemical 1,4-dioxane could cause hyperalgesia through TRPV1 activation [87]. The arachidonic acid metabolites, 20-hydroxyeicosatetraenoic acid could activate TRPV1 and cause neurogenic inflammation in the skin as an endogenous ligand [88]. TRPV1+ nociceptor-microglia interactions can cause persistent visceral pain following colitis [89]. From this, we can conclude that there are still many mysteries about TRPV1 waiting for us to unravel. The exploration of its molecular mechanism and clinical application will be of great significance to human health.

## Strength and limitations

This is a different style from the traditional review to perform a bibliometric and visualized analysis of TRPV1 channel in inflammation field. This method helps us to focus more easily on critical subjects and creative ideas in this field. However, there are existing some limitations for our study. Firstly, we just analyzed the documents in English from WOSCC database, but not in other languages (Chinese, for example) or other database sources. Secondly, we just extracted data from WOSCC database for that many commonly used databases, for example, PubMed, do not provide full text and citation analyses, which are necessary for bibliometric analysis. Thirdly, recent articles could not acquire sufficient attention due to publication time and this may result in missing some new high-quality articles. Despite these limitations, we still believe that our findings can reflect the trend in the field associated with TRPV1 channel and inflammation at a global level. We believe that the limitations will be solved in the future studies.

## Conclusions

In summary, this study analyzed the research of TRPV1 channel in inflammation by the means of bibliometric tools and provided historical retrospect on this field from many aspects including research countries, institutions, authors, journals, citations, trends and hotspots. We hope that this paper can provide some insight and ideas for this research field.

## Supplementary Material

Supplemental Material

## Data Availability

The authors confirm that the data supporting the findings of this study are available within the article and its supplementary materials.
