# Supplementary material for: Bibliometric analysis of recent research on the association between TRPV1 and inflammation
Source: Channels (Austin). 2023 Mar 15;17(1):2189038. doi: 10.1080/19336950.2023.2189038 (PMC10026872; doi:10.1080/19336950.2023.2189038)

|  | Topic | Records |
| --- | --- | --- |
| #1 | "Inflammation" (Topic) and English (Language) | 703144 |
| #2 | "Inflammations" (Topic) and English (Language) | 2373 |
| #3 | "Innate Inflammatory Responses" (Topic) and English (Language) | 191 |
| #4 | "Innate Inflammatory Response" (Topic) and English (Language) | 304 |
| #5 | "Inflammatory Response, Innate" (Topic) and English (Language) | 25 |
| #6 | #1 OR #2 OR #3 OR #4 OR #5 | 704793 |
| #7 | "TRPV1" (Topic) and English (Language) | 8922 |
| #8 | "TRPV-1" (Topic) and English (Language) | 253 |
| #9 | "vanilloid receptor 1" (Topic) and English (Language) | 977 |
| #10 | "transient receptor potential cation channel subfamily V member 1 protein" (Topic) and English (Language) | 1 |
| #11 | "transient receptor potential vanilloid 1" (Topic) and English (Language) | 1755 |
| #12 | "transient receptor potential V1" (Topic) and English (Language) | 90 |
| #13 | "capsaicin receptor" (Topic) and English (Language) | 3242 |
| #14 | #7 OR #8 OR #9 OR #10 OR #11 OR #12 OR #13 | 10801 |
| #15 | #6 AND #14 and Meeting Abstract or Book Chapters or Proceeding Paper or Early Access or Editorial Material or Letter or Retracted Publication or Correction or News Item (Exclude – Document Types) and 2023 or 2022 (Exclude – Publication Years) | 1925 |

Figure S1. The flowchart of TRPV1 channel and inflammation researches.
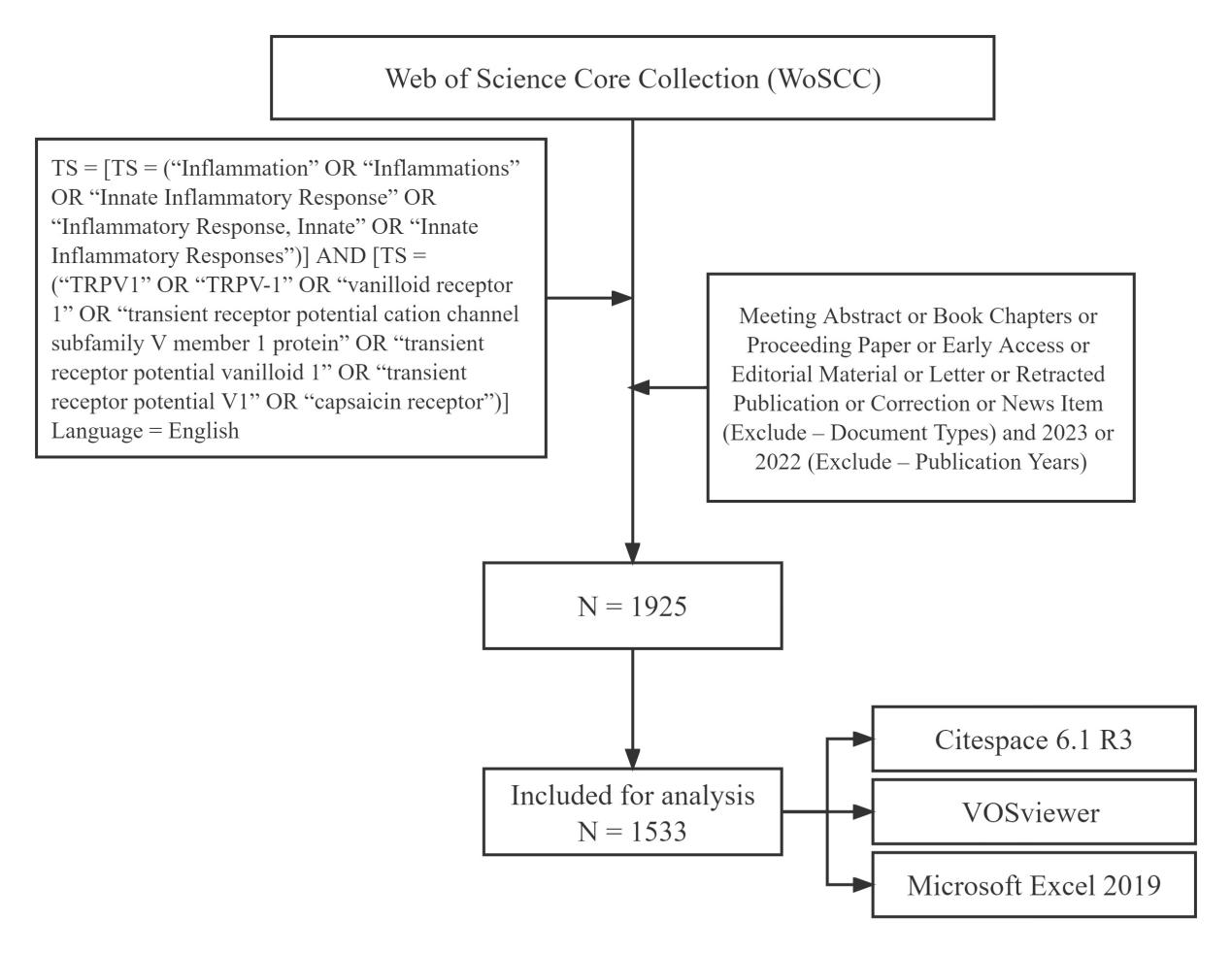

Supplement: Supplemental Material [file KCHL_A_2189038_SM5821.docx]
